# Chlamyphilone, a Novel *Pochonia chlamydosporia* Metabolite with Insecticidal Activity

**DOI:** 10.3390/molecules24040750

**Published:** 2019-02-19

**Authors:** Federica Lacatena, Roberta Marra, Pierluigi Mazzei, Alessandro Piccolo, Maria Cristina Digilio, Massimo Giorgini, Sheridan L. Woo, Pierpaolo Cavallo, Matteo Lorito, Francesco Vinale

**Affiliations:** 1 Dipartimento di Agraria, Università degli Studi di Napoli Federico II, 80055 Portici (NA), Italy; federica.lacatena@unina.it (F.L.); robmarra@unina.it (R.M.); alessandro.piccolo@unina.it (A.P.); digilio@unina.it (M.C.D.); lorito@unina.it (M.L.); 2 Dipartimento di Farmacia (DIFARMA), Università degli Studi di Salerno, 84084 Fisciano (SA), Italy; pmazzei@unisa.it; 3 Centro Interdipartimentale di Ricerca sulla Spettroscopia di Risonanza Magnetica Nucleare, per l’Ambiente, l’Agro-Alimentare ed i Nuovi Materiali (CERMANU), Università degli Studi di Napoli Federico II, 80055 Portici (NA), Italy; 4 Istituto per la Protezione Sostenibile delle Piante, Consiglio Nazionale delle Ricerche (IPSP-CNR), 80055 Portici (NA), Italy; massimo.giorgini@ipsp.cnr.it (M.G.); woo@unina.it (S.L.W.); 5 Dipartimento di Farmacia, Università degli Studi di Napoli Federico II, 80131 Napoli, Italy; 6 Dipartimento di Fisica “E.R. Caianiello”, Università degli Studi di Salerno, 84084 Fisciano (SA), Italy; pcavallo@unisa.it; 7 Istituto Sistemi Complessi, Consiglio Nazionale delle Ricerche (ISC-CNR), 00185 Rome, Italy

**Keywords:** secondary metabolites, beneficial microbes, pea aphid, azaphilones

## Abstract

Metabolites from a collection of selected fungal isolates have been screened for insecticidal activity against the aphid *Acyrthosiphon pisum*. Crude organic extracts of culture filtrates from six fungal isolates (*Paecilomyces lilacinus*, *Pochonia chlamydosporia*, *Penicillium griseofulvum*, *Beauveria bassiana*, *Metarhizium anisopliae* and *Talaromyces pinophilus*) caused mortality of aphids within 72 h after treatment. In this work, bioassay-guided fractionation has been used to characterize the main bioactive metabolites accumulated in fungal extracts. Leucinostatins A, B and D represent the bioactive compounds produced by *P. lilacinus.* From *P. griseofulvum* and *B. bassiana* extracts, griseofulvin and beauvericin have been isolated, respectively; 3-*O*-Methylfunicone and a mixture of destruxins have been found in the active fractions of *T. pinophilum* and *M. anisopliae*, respectively. A novel azaphilone compound, we named chlamyphilone, with significant insecticidal activity, has been isolated from the culture filtrate of *P. chlamydosporia*. Its structure has been determined using extensive spectroscopic methods and chemical derivatization.

## 1. Introduction

Alarm over the impact of pesticides on the environment and human health is increasing year after year, and rigorous pesticide registration procedures have been introduced. Through Directive 2009/128/EC, the European Community has severely restricted the use of synthetic pesticides in plant protection, and the new regulations have reduced the number of chemicals available in agriculture [1]. Novel pesticides, including natural product-based formulations, have been developed to counteract the evolution of resistance among plant pathogen and pest populations [2].

Microorganisms biosynthesize thousands of compounds displaying different biological activities, e.g., acting as antibiotics, therapeutic agents, toxins, hormones, etc. [3]. Thus, the exploitation of the microbial metabolome has become an important area of research to isolate novel natural products potentially useful for agricultural applications [3]. The improvement of screening technologies can be used to extend the search of new microbial active metabolites, i.e., through (i) upgrading of fermentation techniques, (ii) the development of new methods for detection, and (iii) genetic manipulation to create mutants able to produce qualitatively or quantitatively different metabolites [4].

Microorganisms have been extensively screened for antibiotic production; the main producers are fungi and bacteria, particularly the actinomycetes *Streptomyces* spp. [5]. A large number of manuscripts deal with microbial metabolites, which have demonstrated efficacy as crop protection agents, but only a few compounds have been marketed so far [2]. Examples of commercially available microbial metabolites with insecticide activity are: (1) abamectin and anthelmintic produced by the soil-dwelling actinomycete *S. avermitilis*; (2) milbemycin (also known as milbemectin), an insecticide and acaricide from *S. hygroscopicus* subsp. *aureolacrimosus*; (3) polynactins, secondary metabolites from the actinomycete *S. aureus*, isolated and applied as a mixture of tetranactin, trinactin and dynactin; (4) spinosad, a secondary metabolite from the soil actinomycete *Saccharopolyspora spinosa* [2].

The interaction between some fungal strains and the plant establish a molecular cross-talk in which fungal metabolites can act as elicitors that activate the expression of genes involved in plant defence response, and promote the growth of the plant [6]. Identification of new bioactive compounds may be obtained with a good microbial collection or isolating metabolites not expressed under standard laboratory conditions [6,7,8].

In this work, we show the results of a screening aimed to isolate microbial metabolites with insecticidal activity. Various genera of fungi, including some entomopathogenic species, were grown in liquid culture and their major secondary metabolites were investigated. Bioassay-guided fractionation used to isolate the bioactive metabolites allowed the identification of seven known compounds and a new metabolite named chlamyphilone. This compound was fully characterized by spectrometric analysis and chemical derivatization. The fungal metabolites have been screened for insecticidal activity against *Acyrthosiphon pisum* (Hemiptera: Aphididae), the pea aphid, whose high rate of increase makes it a useful model for screening [9].

## 2. Results

The microbes used in the present study includes various genera of fungi identified according to morphological features and molecular analyses (rDNA-ITS and β-tubulin gene sequencing). In particular, 5 isolates belong to the *Trichoderma* genus (*T. tomentosum* F19, *T. asperellum* CINO1, *T. harzianum* M10, *T. harzianum* F53, *T. velutinum* F28) and 4 to *Penicillium* (*P. chrysogenum* F5, *P. decumbens* F29, *P. griseofulvum* F11, *P. restrictum* F55). *Beauveria bassiana* BB1, *Chloridium virescens* F57, *Metarhizium anisopliae* MA3, *Paecilomyces lilacinus* 100379, *Pochonia chlamydosporia* B and *Talaromyces pinophilus* F36CF are present as single species.

All fungi have been grown in static conditions and the culture filtrates were extracted exhaustively with ethyl acetate. The crude extracts were fractionated by column chromatography to provide a total of 79 organic fractions. The extracts and all the fractions were tested at different concentrations on *Acyrthosiphon pisum*. Table 1 reports the percentage of mortality at 72h of the active extracts/fractions obtained from different fungal isolates. Only extracts obtained from *P. lilacinus*, *P. griseofulvum*, *P. chlamydosporia*, *B. bassiana*, *M. anisopliae* and *T. pinophilus* showed significant insecticidal activity (data obtained from the other fungal strains are not showed). Moreover, the bioassay-guided fractionation indicated that only one fraction per strain was responsible of a significant aphid mortality after 72 h exposure (Table 1).

**Figure 1:**
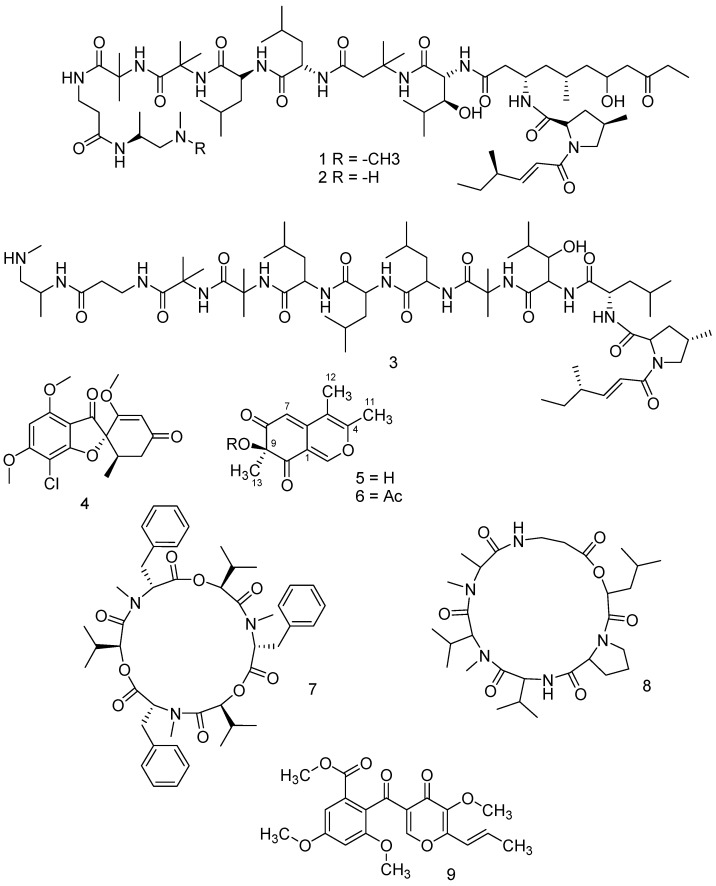
Chemical structures of leucinostatin A, **1**; leucinostatin B, **2**; leucinostatin D, **3**; griseofulvin, **4**; chlamyphilone, **5**; Ac-chlamyphilone, **6**; beauvericin, **7**; dextrusin B2, **8**; 3-*O*-Methylfunicone, **9**.

Chemical characterization of the active fractions was obtained using NMR and/or LC-MS qTOF analyses. Fraction No. 3 of *P. lilacinus* (showing 30% aphid mortality; Table 1) was constituted by a mixture of leucinostatins A, B and D (**1**, **2** and **3,** respectively; Figure 1), with the molecular weights (MW) 1218.634 g/mol, 1204.607 g/mol and 1104.4799 g/mol, respectively (Figure S1).

The mycotoxin griseofulvin (**4** in Figure 1) was determined as the main metabolite of *P. griseofulvum* and was isolated in the active fraction number 7 (showing 73,3% aphid mortality; Table 1). Figure S3 reports the mass spectrum of the isolated compound.

The residue recovered after the organic extraction (350 mg) of *P. chlamydosporia* culture filtrate was subjected to flash column chromatography, eluting with CH_2_Cl_2_/MeOH (90:10 *v*/*v*). Fractions showing similar thin-layer chromatography (TLC) profiles were combined and further purified by using preparative TLC separation (Si gel; CH_2_Cl_2_/MeOH 90:10 *v*/*v*). Both fractions and pure compounds were tested for insecticidal activity. Twelve milligrams of a novel compound, named chlamyphilone (**5** in Figure 1; 5 mg/L), were obtained as a yellow amorphous solid in pure form after TLC (Rf in CH_2_Cl_2_/MeOH 90:10 *v*/*v* = 0.8) and has: [α]^25^_D_ −21.9° (c 1; CH_2_Cl_2_); UV (CH_2_Cl_2_) λ max (log ε) 219 (3.57), 332 (4.23). ^1^H and ^13^C NMR spectral data of chlamyphilone (in CDCl_3_) are presented in Table 2. LC-MS qTOF analysis detected the precursor ions at *m*/*z* 221.0814 (pseudomolecular ion [M + H]^+^); 463.1360 [M_2_ + Na]^+^; 243.0916 [M + Na]^+^; 221.0814 [M + H]^+^ (calcd. 221.0814); 203.1313 [M + H − H_2_O]^+^ (Figure S4). The results obtained by ^13^C NMR (Table 2 and Figure S6) and LC-MS qTOF analyses (Figure S4) were consistent with a compound having a molecular weight of 220.0814, corresponding to the molecular formula C_12_H_12_O_4_ with seven unsaturations.

The UV spectrum showed absorption peaks characteristic of the azaphilone family, which are natural products containing a 6*H*-isochromene-6,8(7*H*)-dione. Six carbon signals at 102.9, 114.4, 115.3, 145.2, 151.7, and 155.1 ppm revealed three double bonds, whereas signals at 196.3 and 196.5 ppm indicated the presence of two ketones. The other four signals in the ^13^C spectrum were all shifted upfield in the 12.7–83.11 ppm range. The DEPT data demonstrated that one of the protons in the molecule is bound to oxygen, and that in the molecule there are three CH_3_, two CH, and seven fully substituted C atoms. The ^1^H-^1^H COSY, HSQC analysis, and the chemical shift evaluation (Table 2) allowed the identification of the structural fragment. In Table 2 all the compound signals are reported.

**Figure 2:**
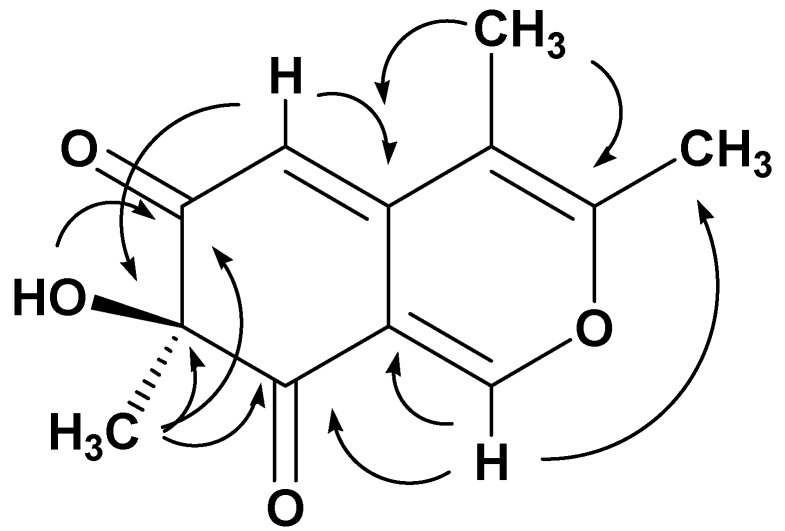
HMBC correlation of chlamyphilone.

The connectivity of the spin systems was deduced by a long-range ^1^H-^13^C heterocorrelated experiment that was obtained with the HMBC (Table 2). In particular, the most relevant HMBC correlations are reported in Figure 2, which implied that the structure of the metabolite **5** is 7-hydroxy-3,4,7-trimethyl-isochromene-6,8-dione. The configuration of chlamyphilone was evident from NOESY experiment. The MM-2 energy calculation (16.0 Kcal/mol) was run to find the most stable conformational model (Figure S12).

To confirm the proposed structure, a sample of chlamyphilone was acetylated using acetic anhydride/pyridine (**6** in Figure 1). The ^1^H-NMR spectrum of the product (acetic acid 3,4,7-trimethyl-6,8-dioxo-7,8-dihydro-6H-isochromen-7-yl ester) contained one acetate resonance, confirming the presence of the hydroxyl group bound to the carbon at 83.1. The ^13^C-NMR spectrum contained an additional signal for the acetyl carbonyl resonance at 170.8 and for the acetate methyl carbon at 18.4 ppm.

Chlamyphilone (**5** in Figure 1) showed the highest insecticidal activity against *Acyrthosiphon pisum* with a median Lethal Dose (LD50) of 175 μg/mL and a minimal inhibitory concentration (MIC) of 150 μg/mL (Table 3).

The insecticidal activity of the *B. bassiana* strain used in the present work was mainly dependent on the presence of beauvericin (**7** in Figure 1). This metabolite is a cyclohexadepsipeptide mycotoxin with the molecular formula C_45_H_57_N_3_O_9_ (Figure S13).

From *M. anisopliae* extract, destruxin B2 has been characterized (**8** in Figure 1). Destruxins are cyclic hexadepsipeptides composed of an α-hydroxy acid and five amino acid residues. Individual destruxins differ in terms of the hydroxy acid, *N*-methylation, and R group of the amino acid residues. Destruxin B2 has the molecular formula C_29_H_49_N_5_O_7_, as revealed by the MS spectrum (Figure S14).

Finally, the major active metabolite produced by *T. pinophilus* strain F36CF was 3-*O*-methylfunicone (OMF—**9** in Figure 1), a well-known γ-pyrone derivative, previously isolated from *Talaromyces* spp.

## 3. Discussion

In this work, a functional screening of fungal strains, preliminarily selected because of their ability to synthetize active metabolites, has been carried out. Bioassay-guided fractionation has been used to isolate microbial metabolites with insecticidal activity against the pea aphid *A. pisum* (Hemiptera: Aphididae), long used as a model to study plant‒insect interactions [9]. This aphid is an important pest of several plants, is a phloem-feeding insect determining direct negative effects on plants in terms of nutritive subtraction and injection of toxic saliva. Moreover, it has been involved in the transmission of diverse plant viruses [9].

The active fraction obtained from *P. lilacinus* was constituted by a mixture of leucinostatins A, B, and D. Leucinostatins are peptides containing an unsaturated fatty acid, nine amino acid residues, and a basic component joined together by amide linkages [10]. These antibiotic metabolites also showed antitumor and nematocidal activities and an uncoupling effect on rat liver mitochondrial function [10,11]. To the best of our knowledge, our work is the first report on the insecticidal activity of leucinostatins against *A. pisum*.

Griseofulvin was determined to be the main metabolite of *P. griseofulvum*. The insecticidal activity of this metabolite has been previously demonstrated in the corn earworm, *Helicoverpa zea* Boddie, and the fall armyworm, *Spodoptera frugiperda* (J. E. Smith), by oral administration in an artificial diet (250 ppm) [12,13,14]. In the case of soft-tegument aphids like *A. pisum*, the activity is probably topical as oral administration may be excluded because of their peculiar feeding pattern. In fact, aphids use their piercing, sucking mouthparts to feed on plant fluid [9].

The extract of *P. chlamydosporia* culture filtrate yielded 12 mg of a novel compound named chlamyphilone. This metabolite belongs to the class of azaphilones, which contain a 6*H*-isochromene-6,8(7*H*)-dione or an isoquinoline-6,8(2*H*,7*H*)-dione skeleton (and its substituted derivatives thereof). This is the first report on the isolation of an azaphilone metabolite from *P. chlamydosporia*. A similar azaphilone, named myxostiol, with plant growth regulating activity, has been previously isolated from *Myxotrichum stipitatum* [15]. Over 170 different azaphilone compounds, classified into 10 structural groups, have been isolated from fungi belonging to 23 genera [16]. Azaphilones display a wide range of biological activities, such as antimicrobial, antifungal, antiviral, antioxidant, cytotoxic, nematocidal, and anti-inflammatory [16]. Many of these properties may be explained by the reactions of azaphilones with amino groups, such as those found in amino acids, proteins, and nucleic acids, resulting in the formation of vinylogous c-pyridones [16,17,18]. To the best of our knowledge, this is the first evidence of the insecticidal activity of a metabolite belonging to the class of azaphilones. Recently, the involvement of small molecules (e.g., aurovertins) in the interaction between nematodes and *P. chlamydosporia* has been demonstrated [19].

An active beauvericin has been extracted from culture filtrate of *B. bassiana*. This metabolite is a mycotoxin whose insecticidal properties have been previously reported (e.g., against the wheat aphid *Schizaphis graminum* at 0.5 mg/mL) [20,21]. This molecule contains three residues, each with D-2-hydroxyisovaleric acid (Hiv) and L-*N*-methylphenylalanine linked alternately [21].

Destruxin B2 is the active molecule isolated from *M. anisopliae* extract. Destruxins exhibited a wide variety of biological activities, but are best known for their insecticidal and phytotoxic properties [22,23]. However, the effect of destruxin B2 against *Acyrthosiphon pisum* is reported here for the first time.

*T. pinophilus* strain F36CF produced 3-*O*-methylfunicone. This compound was downregulated in the presence of *T. harzianum* M10 [8], exhibited notable antibiotic and antitumor properties, and recently exhibited an insecticidal effect, thus expanding the biological activities of this compound [24]. Recently, talarodiolide, a new 12-membered macrodiolide, was isolated and characterized from the culture filtrate of a *T. pinophilus* strain [25]. This metabolite did not show insecticidal activity, highlighting that the strain collection is important to select the best producers of bioactive compounds.

## 4. Materials and Methods

**Fungal strains.** The microbes used in the present study were present in the fungal collection at Department of Agricultural Sciences, University of Naples Federico II (UNINA Collection) or isolated from different sources (Table 4).

The fungi were identified according to morphological features and molecular analyses. Briefly, an amount of 10^6^ spores/mL was inoculated in a 250-mL Erlenmeyer Flasks containing 100 mL of sterile Potato Dextrose Broth (PDB, HIMEDIA, Mumbai, India). Each flask was incubated at 25 °C in an orbital shaker (150 r.p.m.) and fungi were left to grow for seven days. Mycelium was recovered, ground to a powder in liquid nitrogen, and used to perform the DNA extraction. Genetic analysis was carried out through PCR and sequencing of the rDNA Internal Transcribed Spacer (ITS) and β-tubulin gene, using the most common primers to identify fungal strains: ITS1 and ITS4 [26], as well as tub2 and BenA [27]. The PCR products were subjected to gel electrophoresis, excised, and sequenced [26,27]. Analysis of the ITS gave 99% of identity with GenBank sequences of the fungi reported in Table 4, confirming the identity of the microbes.

**Secondary metabolites production.** The fungi were maintained on potato dextrose agar (PDA, HIMEDIA) at room temperature and sub-cultured bimonthly. Liquid cultures were prepared in 5000 L-Erlenmeyer flasks containing 1 L of PDB and inoculated with five mycelial plugs (7 mm^2^) from a fresh PDA culture of each fungal strain. After 30 days of incubation at 25 °C in static conditions, the cultures were filtered through filter paper (Whatman No. 4) and the filtrate was analyzed by LC-MS qTOF [28].

**Extraction and isolation of fungal secondary metabolites.** The culture filtrates were acidified to pH 4 with 5 M HCl and extracted exhaustively with ethyl acetate (EtOAc). The combined organic extracts were dried (Na_2_SO_4_) and solvent eliminated under reduced pressure at 35 °C (Rotavapor RV 10 IKA^®^ - Werke GmbH & Co. KG, Staufen, Germany). The residues recovered were fractionated by column chromatography (silica gel; 200 g) eluted with different eluents (Table 5) and the homogeneous fractions were collected as reported in Table 5. For thin layer chromatography (TLC) the following solvents were used: dichloromethane (CH_2_Cl_2_)/methanol (MeOH) 90:10; 80:20 (*v*/*v*), chloroform (CHCl_3_)/MeOH 90:10; 80:20 (*v*/*v*); EtOAc/petroleum ether 90:10; 80:20 (*v*/*v*) [28]. All fractions were tested for insecticidal activity against *A. pisum*, and, where necessary, further purified by preparative TLC (Table 5).

**General experimental procedures.** The isolated molecules were solubilised in 700 µL of deuterated chloroform (99.8% CDCl_3_ – Sigma-Aldrich, Darmstadt, Germany) and transferred into a stoppered NMR tube (5 mm, 7′′, 507-HP-7, NORELL, Morganton, NC, USA) where remaining void volume was gently degassed by a N_2_ flux. Proton and carbon solvent signals were used as reference to calibrate both ^1^H and ^13^C frequency axes. A 400 MHz Bruker Avance spectrometer (Bruker Co., Billerica, MA, USA), equipped with a 5 mm Bruker Broad Band Inverse probe (BBI), working at the ^1^H and ^13^C frequencies of 400.13 and 100.61 MHz, respectively, was used for the NMR measurements (at 25 ±1 °C).

Monodimensional ^1^H and ^13^C acquisitions were conducted as follows: proton spectra were acquired with 2 s of thermal equilibrium delay (number of scans = 64), a 90° pulse length 7.7 µs, 50 transients and 16 ppm (6410.2 Hz) as spectral widths, whereas proton-decoupled carbon acquisitions were executed by both inverse-gated and DEPT 135° pulse sequences, adopting 7 and 5 s of equilibrium delay, 12,500 and 2400 transients, respectively, and a spectral width of 250 ppm (25.152 KHz). A time domain of 32,768 points was adopted for all cited mono-dimensional experiments. Homo-nuclear ^1^H-^1^H COSY (COrrelation SpectroscopY), TOCSY (TOtal Correlation SpectroscopY), NOESY (Nuclear Overhauser Enhancement SpectroscopY), and hetero-nuclear ^1^H–^13^C HSQC (Hetero-nuclear Single-Quantum Correlation) and HMBC (Hetero-nuclear Multiple Bond Coherence) experiments (2D) were used for structural identification of metabolites. 2D homo- and heteronuclear spectra experiments were acquired with 48 and 80 scans, respectively, 16 dummy scans, a time domain of 2k points (F2) and 256 experiments (F1). TOCSY and NOESY experiments were conducted with a mixing time of 80 and 1000 ms, respectively, while HSQC and HMBC experiments were optimized for 145 Hz short and 6.5 Hz long range J_CH_ couplings, respectively. All executed 2D experiments were gradient enhanced, except for the TOCSY acquisition. A Qsine weighting function associated to a magnitude mode was used to process NOESY spectrum with the purpose to emphasize the weak cross-peaks and minimize the noise artefacts. The free induction decay (FID) of mono-dimensional spectra was multiplied by an exponential factor corresponding to 0.1 Hz, for ^1^H and ^13^C acquisitions, and to 1 Hz for DEPT 135° experiment. All above mentioned spectra were baseline corrected and processed by using Bruker Topspin Software (v.4.0.2).

LC-MS/MS Q-TOF analysis were done on an Agilent HP 1260 Infinity Series liquid chromatograph equipped with a DAD system (Agilent Technologies, Santa Clara, CA, USA) coupled to a Q-TOF mass spectrometer model G6540B (Agilent Technologies). Separations were performed on a Zorbax Eclips Plus C18 column, 4.6 × 100 mm, with 3.5 µm particles (Agilent Technologies). The analyses were done at a constant temperature of 37 °C and using a linear gradient system composed of A: 0.1% (*v*/*v*) formic acid in water, and B: 0.1% (*v*/*v*) formic acid in acetonitrile. The flow was 0.6 mL/min, 95% A graduating to 100% B in 12 min, 100% B 12-15 min, 95% A 15-17 and equilibrating 95% A 17–20 min. The UV spectra were collected by DAD every 0.4 s from 190 to 750 nm with a resolution of 2 nm. The MS system was equipped with a Dual Electrospray Ionization (ESI) source and operated with Agilent MassHunter Data Acquisition Software, rev. B.05.01 in the positive or negative mode. Mass spectra were recorded in the range *m*/*z* 100–1600 as centroid spectra, with 3 scans per second. Two reference mass compounds were used to perform the real-time lock mass correction, purine (C_5_H_4_N_4_ at *m*/*z* 121.050873, 10 µmol/L) and hexakis (1H,1H, 3H-tetrafluoropentoxy)-phosphazene (C_18_H_18_O_6_N_3_P_3_F_24_ at *m*/*z* 922.009798, 2 µmol/L). The capillary was maintained at 4000 V, fragmentor voltage at 180 V, cone 1 (skimmer 1) at 45 V, Oct RFV at 750 V. Gas temperature was 350 °C during the run at 11 L/min, and the nebulizer was set at 45 psig. The injected sample volume was 5 µL.

MS/MS spectra were simultaneously recorded for confirmation purposes of new compounds, using the operating parameters described above, unless otherwise stated. The instrument was operated in the range *m*/*z* 100-1000, recording two spectra per second in targeted acquisition mode (targeted mass: 244.1197, Z = 1, RT 5.88 ±0.5 min). The sample collision energy was set to 20 V.

LC-MS data were evaluated using MassHunter Qualitative Analysis Software B.06.00 and compared to known compounds included in an in-house database. The database contains information of about 4000 known secondary metabolites isolated from more than 80 different fungal genera, and recorded according to their name, molecular formula, monoisotopic mass and producing organism. Positive identifications of fungal metabolites were reported if the compound was detected with a mass error below 10 ppm and with a sufficient score. Standards were used to confirm the chemical identifications.

UV spectra were recorded with a V-730 UV-Visible Spectrophotometer JASCO (Mary’s Court, Easton, MD, USA). Column chromatography was performed using silica gel (Merck silica gel 60 GF_254_; Merck, Darmstadt, Germany), and TLC with glass pre-coated silica gel GF_254_ plates (Merck Kieselgel 60 GF_254_, 0.25 mm). The compounds were detected on TLC plates using UV light (254 or 366 nm) and/or by dipping the plates in a 5% (*v*/*v*) H_2_SO_4_ solution in ethanol followed by heating at 110 °C for 10 min [28,29].

**Acetylation of chlamyphilone.** Acetic anhydride (40 µL) was added to chlamyphilone (**1**, 2.5 mg), dissolved in dry pyridine (80 µL 2.5 mg), and the residue was purified by preparative TLC on silica gel (petroleum ether/aceton, 20:80, *v*/*v*) to yield the acetyl derivative **6** (1.4 mg, 49%) [29]. The reaction was monitored by LC-MS analysis (Figure S1).

**In vivo insecticidal assay.** Insecticidal activity of organic extracts, each column fraction and pure metabolites were tested against the pea aphid *Acyrthosiphon pisum*. Aphids were reared on *Vicia faba* var. *aguadulce* in a growth chamber (20 ± 1 °C, 70% RH, 18 h light/6 h dark photoperiod). Small populations were synchronized to obtain newborn nymphs every 24 h. Third-instar nymphs were dipped in the assay solution for 10 s and put on a paper towel to dry. Then, each insect was carefully transferred by a soft paintbrush on a leaf plug [30,31]. Twenty treated (or untreated—solvent control) 3rd-instar nymphs were placed on two circular (35 mm diameter) leaf plugs on 2% water agar layer in a Petri dish, in order to keep the leaf turgid. Plates were incubated at the climatic conditions described above and the number of dead aphids was assessed 24, 48, and 72 h after treatments. Extracts, fractions, and pure compounds were tested at 500, 400, 300, 200, 100, and 50 μg/mL. Each metabolite solution was also tested for phytotoxicity on *V. faba*. The aphid mortality has been calculated in each treatment as the mean value ± standard deviation, in three replicates for each time point (24, 48, and 72 h). The experiments were repeated at least twice.

**Statistical analysis.** Data analysis was performed with SPSS 11.0 software (Statistics for Windows Version 24.0, IBM Corp., Armonk, NY, USA), and statistical analysis was done using one-way analysis of variance (ANOVA). The Least Significant Difference (LSD) post hoc test with *p* < 0.05 was used to analyse the multiple comparisons. The mean values between different treatments at the same time point and the mean values between the same treatments at different time points were compared.

## 5. Conclusions

In this work six known metabolites with activity against the pea aphid *A. pisum* have been isolated from culture filtrates of selected fungal strains; for some of them this is the first report of insecticidal activity (Table S1). Moreover, a novel anti-aphid natural product, named chlamyphilone, has been isolated form the culture filtrate of *P. chlamydosporia* and fully characterized. The isolation of natural products with beneficial effects for plants may help to formulate novel secondary metabolites–based biopesticides, which represent promising alternatives to synthetic chemicals in agriculture. Further investigations should optimize extraction protocols and define environmental parameters that can affect the commercial formulations based on these natural compounds.

## Figures and Tables

**Table 1:** Insecticidal activity of fungal organic extracts and active fractions as mortality (%) of the pea aphid *Acyrthosiphon pisum* at 72 h after exposure. Different lower letters refer to significant differences (*p* < 0.05) among treatments at the same incubation time. Extracts from *T. tomentosum* F19, *T. asperellum* CINO1, *T. harzianum* M10, *T. harzianum* F53, *T. velutinum* F28, *P. chrysogenum* F5, *P. decumbens* F29, *P. restrictum* F55 and *C. virescens* F57 did not show significant insecticidal activity.

Microbes	Aphid Mortality (%) at 72 h after Exposure to Organic Extract (10 mg/mL)	Standard Deviation	Active Fraction (Number)	Aphid Mortality (%) at 72 h after Exposure to Active Fraction (0.5 mg/mL)	Standard Deviation
*Paecilomyces lilacinus*	6.0 ^a^	±3.6	3	30.0 ^a^	±18.0
*Penicillium griseofulvum*	63.3 ^b^	±21.0	7	73.3 ^b^	±8.0
*Pochonia chlamydosporia*	50.0 ^b^	±13.0	2	100.0 ^c^	±0.0
*Beuveria bassiana*	40.0 ^b^	±17.0	3	40.0 ^a^	±22.9
*Metarhizium anisopliae*	53.3 ^b^	±5.7	5	60.0 ^a^	±10.0
*Talaromyces pinophilus*	65.0 ^b^	±15.0	2	48.3 ^a^	±5.8

**Table 2:** ^1^H and ^13^C NMR spectral data of chlamyphilone (in CDCl_3_).

Position	δ_C_ Mult.	δ_H_ (*J* in Hz)	HMBC *
1	115.3 qC		
2	151.7 CH	7.91 d (1.37)	1, 10, 11
4	155.1 qC		
5	114.4 qC		
6	145.2 qC		
7	102.9 CH	5.55 d (1.37)	6, 9
8	196.3 qC		
9	83.1 qC		
10	196.5 qC		
11	17.5 CH_3_	2.26 d (0.6)	2
12	12.7 CH_3_	1.93 d (0.6)	4, 6
13	28.6 CH_3_	1.55 s	9, 10, 8
OH		3.9	8

* All correlations represent 2 or 3 bond couplings. Abbreviation, s: singlet, d: doublet, t: triplet, dd: doublet of doublets.

**Table 3:** Insecticidal activity of chlamyphilone **5** at different concentrations. Treatments were evaluated as mortality (%) of the pea aphid *Acyrthosiphon pisum* at 72 h after exposure to **5**.

Concentrations (μg/mL)	Aphid Mortality (%)	Standard Deviation
500	100.0	±0.0
200	100.0	±0.0
190	96.7	±5.8
180	51.7	±2.9
170	30.0	±5.0
160	8.3	±2.9
150	3.3	±2.8

**Table 4:** Fungal strains used in the present study for the isolation of metabolites with insecticidal activity against the pea aphid *A. pisum*.

Microbes	Strain	Division	Class	Order	Family	Habitat/Source *
*Beauveria bassiana*	BB1	*Ascomycota*	*Sordariomycetes*	*Hypocreales*	*Cordycipitaceae*	UNINA Collection
*Chloridium virescens*	F57	*Ascomycota*	*Sordariomycetes*	*Chaetosphaeriales*	*Chaetosphaeriaceae*	Pasture
*Metarhizium anisopliae*	MA3	*Ascomycota*	*Sordariomycetes*	*Hypocreales*	*Clavicipitaceae*	UNINA Collection
*Paecilomyces lilacinus*	100379	*Ascomycota*	*Eurotiomycetes*	*Eurotiales*	*Trichocomaceae*	UNINA Collection
*Penicillium chrysogenum*	F5	*Ascomycota*	*Eurotiomycetes*	*Eurotiales*	*Trichocomaceae*	Mediterranean area
*Penicillium decumbens*	F29	*Ascomycota*	*Eurotiomycetes*	*Eurotiales*	*Trichocomaceae*	Mediterranean area
*Penicillium griseofulvum*	F11	*Ascomycota*	*Eurotiomycetes*	*Eurotiales*	*Trichocomaceae*	Mediterranean area
*Penicillium restrictum*	F55	*Ascomycota*	*Eurotiomycetes*	*Eurotiales*	*Trichocomaceae*	Mediterranean area
*Pochonia chlamydosporia*	B	*Ascomycota*	*Sordariomycetes*	*Hypocreales*	*Clavicipitaceae*	UNINA Collection
*Talaromyces pinophilus*	F36CF	*Ascomycota*	*Eurotiomycetes*	*Eurotiales*	*Trichocomaceae*	*Arbutus unedo*
*Trichoderma tomentosum*	F19	*Ascomycota*	*Sordariomycetes*	*Hypocreales*	*Hypocreaceae*	Woodland (Oak)
*Trichoderma asperellum*	CINO1	*Ascomycota*	*Sordariomycetes*	*Hypocreales*	*Hypocreaceae*	UNINA Collection
*Trichoderma harzianum*	M10	*Ascomycota*	*Sordariomycetes*	*Hypocreales*	*Hypocreaceae*	UNINA Collection
*Trichoderma harzianum*	F53	*Ascomycota*	*Sordariomycetes*	*Hypocreales*	*Hypocreaceae*	Mediterranean area
*Trichoderma velutinum*	F28	*Ascomycota*	*Sordariomycetes*	*Hypocreales*	*Hypocreaceae*	Woodland (Oak)

* UNINA Collection: fungal collection available at the Department of Agricultural Sciences/University of Naples Federico II, Naples, Italy.

**Table 5:** List of solvents used for chromatographic separations (column chromatography or preparative TLC) and total number of homogeneous collected fractions.

Fungal Source	Eluent Used for Column Chromatography and Preparative TLC *	Total Number of Homogeneous Fractions
*Paecilomyces lilacinus*	EtOAc/petroleum ether (90:10, v:v)	5
*Penicillium griseofulvum*	EtOAc/petroleum ether (90:10, v:v)	8
*Pochonia chlamydosporia*	CH_2_Cl_2_/MeOH (90:10, v:v); fraction No. 4 out of 5 was further purified by preparative TLC on silica gel with CH_2_Cl_2_/MeOH (90:10, v:v)	5
*Beauveria bassiana*	EtOAc/petroleum ether (90:10, v:v)	5
*Metarhizium anisopliae*	EtOAc/petroleum ether (90:10, v:v)	7
*Talaromyces pinophilus*	CH_2_Cl_2_/MeOH (98:2, v:v); Fraction No. 2 out of 7 was further purified by preparative TLC with CH_2_Cl_2_/MeOH (98:2, v:v)	7

* EtOAc: ethyl acetate; CH_2_Cl_2_: Dichloromethane; MeOH: Methanol.

## Supplementary Materials

The following are available online, Figures S1–S11 and S13‒S14: NMR and MS spectra of compounds. Figure S12: Conformational model of the new compound named chlamyphilone. Table S1: Biological activity of the isolated compounds.
