# Supplementary material for: Chlamyphilone, a Novel Pochonia chlamydosporia Metabolite with Insecticidal Activity
Source: Molecules. 2019 Feb 19;24(4):750. doi: 10.3390/molecules24040750 (PMC6412625; doi:10.3390/molecules24040750)
Supplement: Supplementary file 1 [file molecules-24-00750-s001.pdf]

SUPPLEMENTARY MATERIALS

Chlamyphilone, a Novel *Pochonia chlamydosporia* Metabolite with Insecticidal Activity

Federica Lacatena [1,†], Roberta Marra [1,†], Pierluigi Mazzei [2,3], Alessandro Piccolo [1,3], Maria Cristina Digilio [1], Massimo Giorgini [4], Sheridan L. Woo [4,5], Pierpaolo Cavallo [6,7], Matteo Lorito [1,4] and Francesco Vinale [1,4,*]

[1] Dipartimento di Agraria, Università degli Studi di Napoli Federico II, 80055 Portici (NA), Italy; federica.lacatena@unina.it (F.L.); robmarra@unina.it (R.M.); alessandro.piccolo@unina.it (A.P.); digilio@unina.it (M.C.D.); lorito@unina.it (M.L.)

[2] Dipartimento di Farmacia (DIFARMA), Università degli Studi di Salerno, 84084 Fisciano (SA), Italy; pmazzei@unisa.it

[3] Centro Interdipartimentale di Ricerca sulla Spettroscopia di Risonanza Magnetica Nucleare, per l'Ambiente, l'Agro-Alimentare ed i Nuovi Materiali (CERMANU), Università degli Studi di Napoli Federico II, 80055 Portici (NA), Italy

[4] Istituto per la Protezione Sostenibile delle Piante, Consiglio Nazionale delle Ricerche (IPSP-CNR), 80055 Portici (NA), Italy; massimo.giorgini@ipsp.cnr.it

[5] Dipartimento di Farmacia, Università degli Studi di Napoli Federico II, 80131 Napoli, Italy; woo@unina.it

[6] Dipartimento di Fisica "E.R. Caianiello", Università degli Studi di Salerno, 84084 Fisciano (SA), Italy; pcavallo@unisa.it

[7] Istituto Sistemi Complessi, Consiglio Nazionale delle Ricerche (ISC-CNR), Rome, Italy;

* Correspondence: francesco.vinale@ipsp.cnr.it; Tel.: +39-081-253-9338

† These authors contributed equally to this work.

Figures S1–S11 and S13-S14: NMR and MS spectra of compounds. Figure S12: Conformational model of the new compound named chlamyphilone. Table S1: Biological activity of the isolated compounds.

Contents

Figure S1. Mass spectrum of acetyl chlamyphilone; m/z 263.1409,63 = [M+H]+.

Figure S2. Mass spectrum of fraction 3 obtained from *P. lilacinus* corresponding to a mixture of Leucinostatins (A, B e D); m/z 1219,63 = [M+H]+; m/z 1241,63 = [M+Na]+; m/z 1201,63 = [M+ H–H_2O]+ (Leucinostatin A); m/z 1205,60 = [M+H]+; m/z 1227,6 = [M+Na]+; m/z 1187,6 = [M+ H–H_2O]+ (Leucinostatin B); m/z 1105,47 = [M+H]+; m/z 1127,47 = [M+Na]+; m/z 1087,47 = [M+ H–H_2O]+ (Leucinostatin D).

Figure S3. Mass spectrum of fraction 7 obtained from organic extracts of *P. griseofulvum* m/z 335,1234 = $[M + H - H_2O]^+$; m/z 353,0793 = $[M+H]^+$; m/z 375,0793 = $[M + Na]^+$; m/z 727,1318 = $[M_2 + Na]^+$.

Figure S4. Mass (A) and fragmentation spectra (B) of chlamyphilone.

Figure S5. ^{1}H NMR of chlamyphilone.

Figure S6. ^{13}C NMR of chlamyphilone.

Figure S7. DEPT 135 of chlamyphilone.

Figure S8. ^{1}H-^{1}H COSY of chlamyphilone.

Figure S9. ^{1}H-^{13}C HSQC of chlamyphilone.

Figure S10. ^{1}H-^{13}C HMBC of chlamyphilone.

Figure S11. NOESYof chlamyphilone.

Figure S12. Conformational model of chlamyphilone.

Figure S13. Mass spectrum of fraction 3 from *B. bassiana* corresponding to beauvericin; m/z 784,96 = $[M+H]^+$; m/z 766,96 = $[M+ H–H_2O]^+$; m/z 806,96 = $[M+Na]^+$; m/z 1590,92 = $[M_2 + Na]^+$.

Figure S14. Mass spectrum of Destruxin B2; m/z 579,2926 = $[M+H]^+$; m/z 561,3420 = $[M+H-H_2O]^+$; m/z 617,2034 = $[M+K]^+$.

Table S1. Biological activity of the isolated compounds.

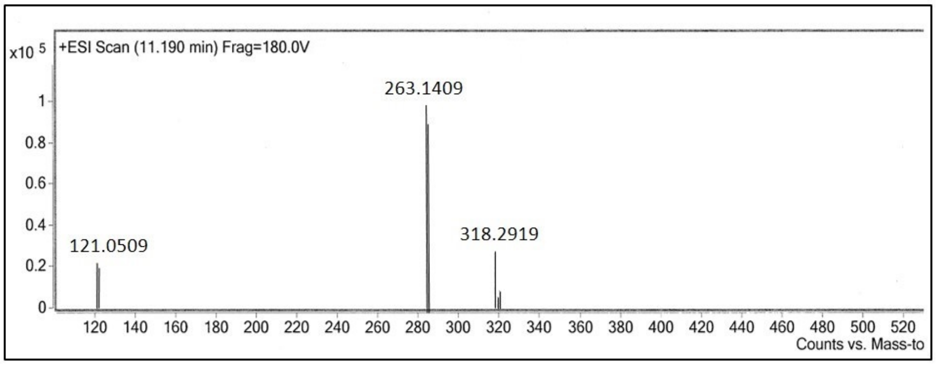

Figure S1. Mass spectrum of acetyl chlamyphilone; m/z 263.1409,63 = [M+H]+.

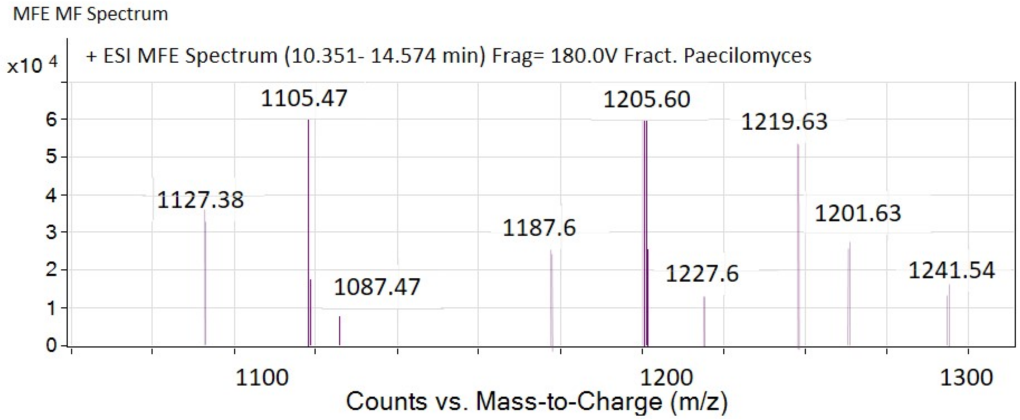

Figure S2. Mass spectrum of fraction 3 obtained from *P. lilacinus* corresponding to a mixture of Leucinostatins (A, B e D); m/z 1219,63 = [M+H]+; m/z 1241,63 = [M+Na]+; m/z 1201,63 = [M+ H–H_2O]+ (Leucinostatin A); m/z 1205,60 = [M+H]+; m/z 1227,6 = [M+Na]+; m/z 1187,6 = [M+ H–H_2O]+ (Leucinostatin B); m/z 1105,47 = [M+H]+; m/z 1127,47 = [M+Na]+; m/z 1087,47 = [M+ H–H_2O]+ (Leucinostatin D).

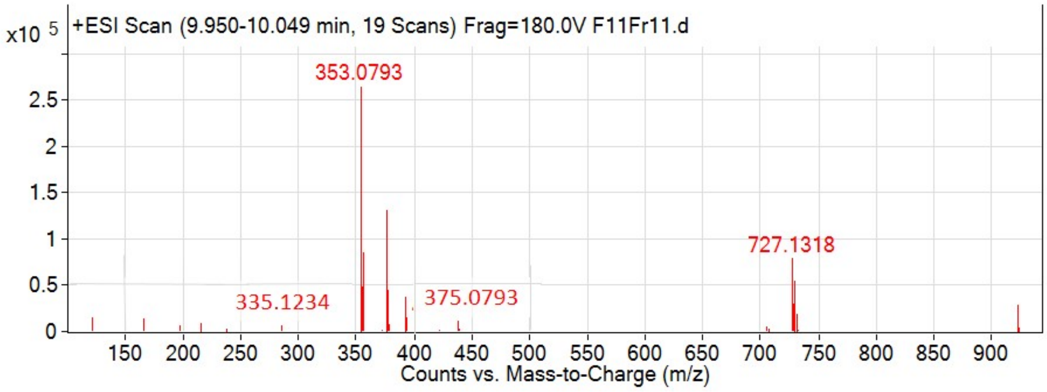

Figure S3. Mass spectrum of fraction 7 obtained from organic extracts of *P. griseofulvum* m/z 335,1234 = [M + H – H_2O]$^+$; m/z 353,0793 = [M+H]$^+$; m/z 375,0793 = [M + Na]$^+$; m/z 727,1318 = [M_2 + Na]$^+$.

(A)

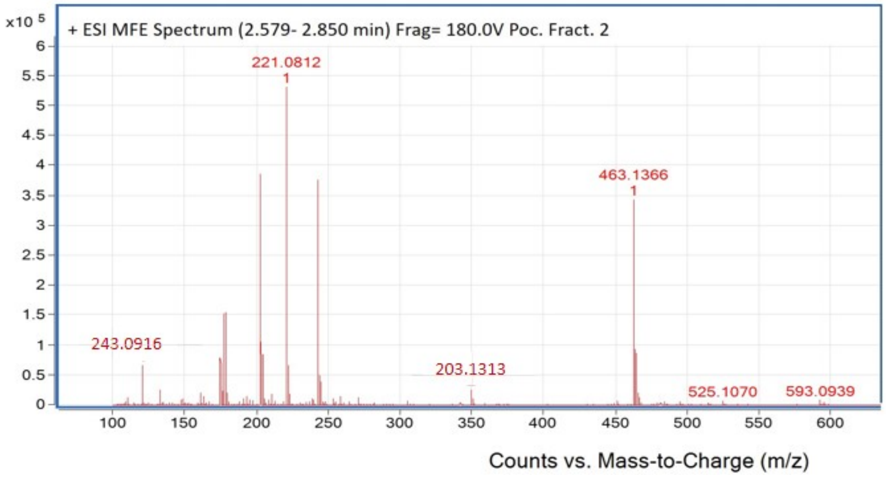

(B)

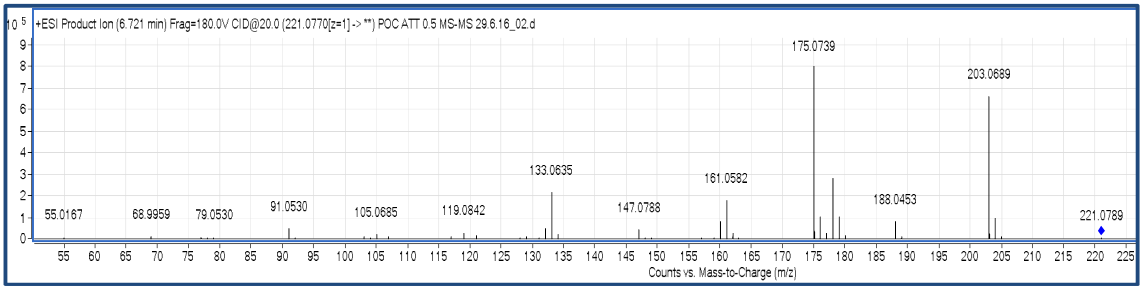

Figure S4. Mass (A) and fragmentation spectra (B) of chlamyphilone.

188 = 203.1313 $[M+H-H_2O]^+$ - $-CH_3$

175 = 203.1313 $[M+H-H_2O]^+$ - $-CH_3 – CH$

161 = 203.1313 $[M+H-H_2O]^+$ - $-CH_3 – CH – CH_2$

147 = 203.1313 $[M+H-H_2O]^+$ - $-CH_3 – CH – CH_2 – CH_2$

133 = 203.1313 $[M+H-H_2O]^+$ - $-CH_3 – CH – CH_2 – CH_2 – CH_2$

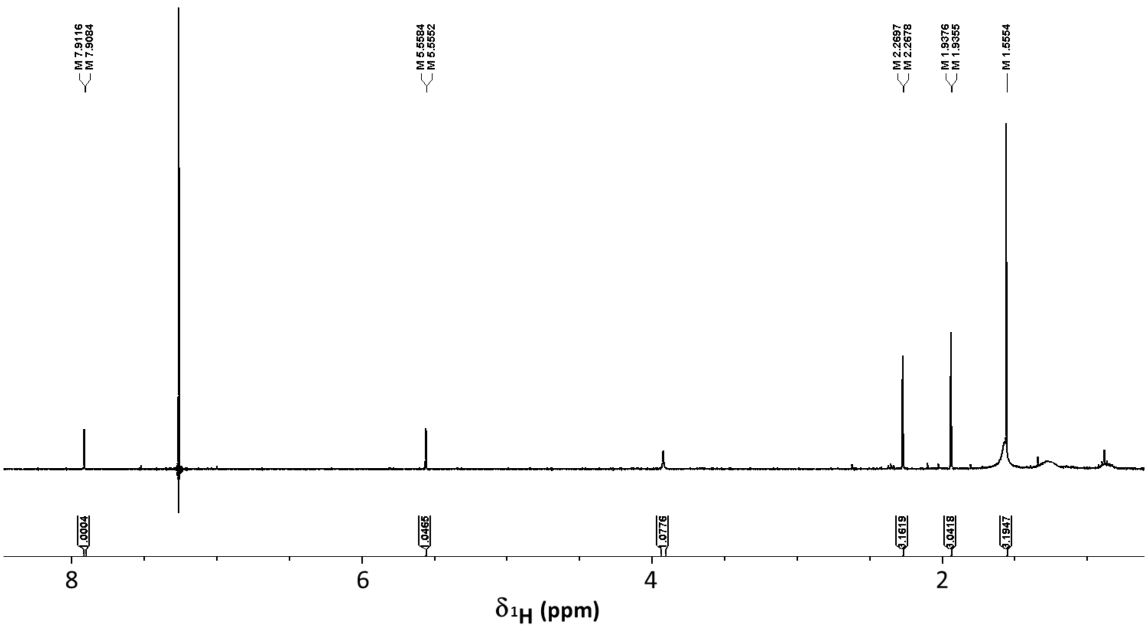

Figure S5. ¹H NMR of chlamyphilone.

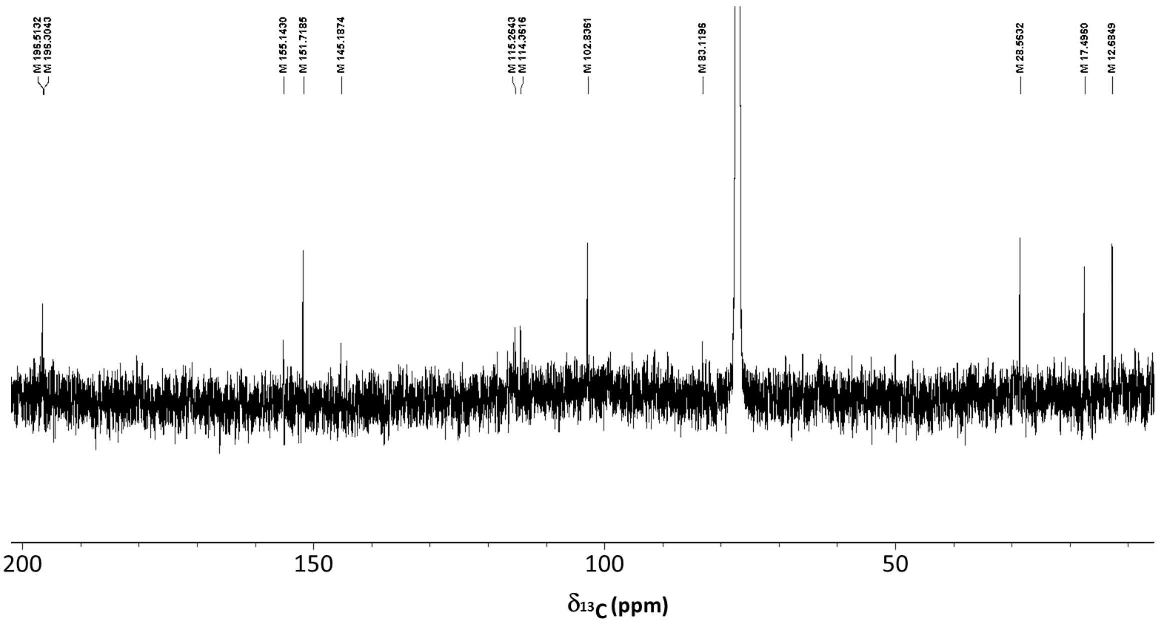

Figure S6. ^{13}C NMR of chlamyphilone.

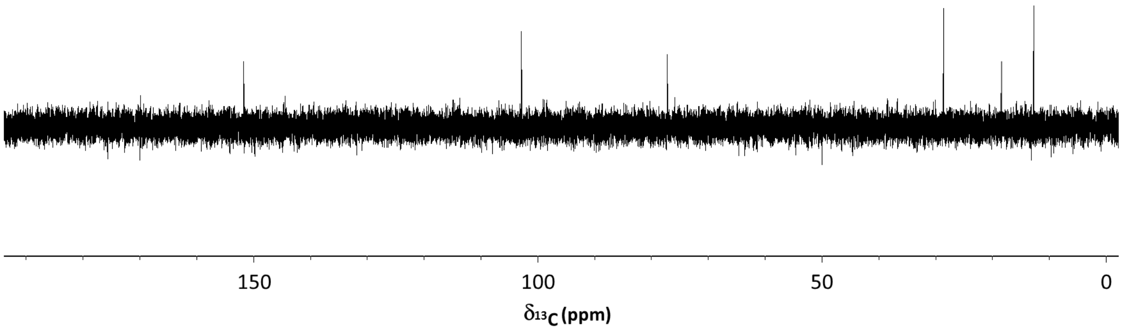

Figure S7. DEPT 135 of chlamyphilone.

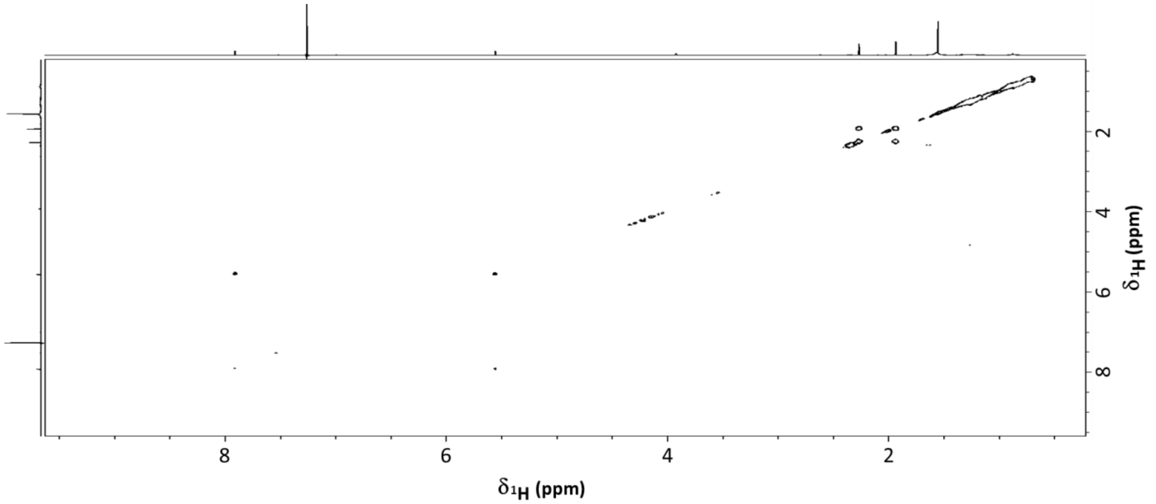

Figure S8. ^{1}H-^{1}H COSY of chlamyphilone.

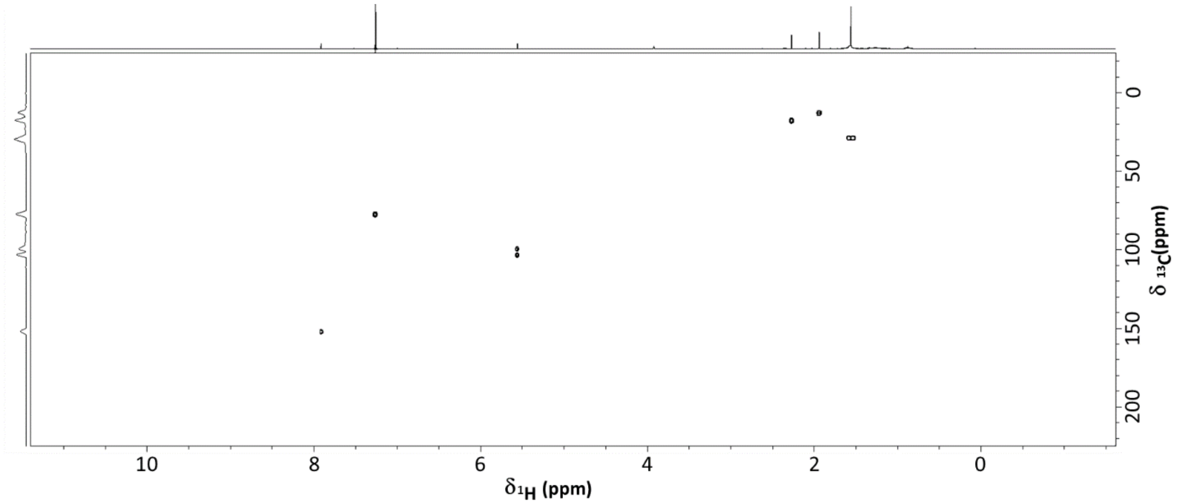

Figure S9. ^{1}H-^{13}C HSQC of chlamyphilone.

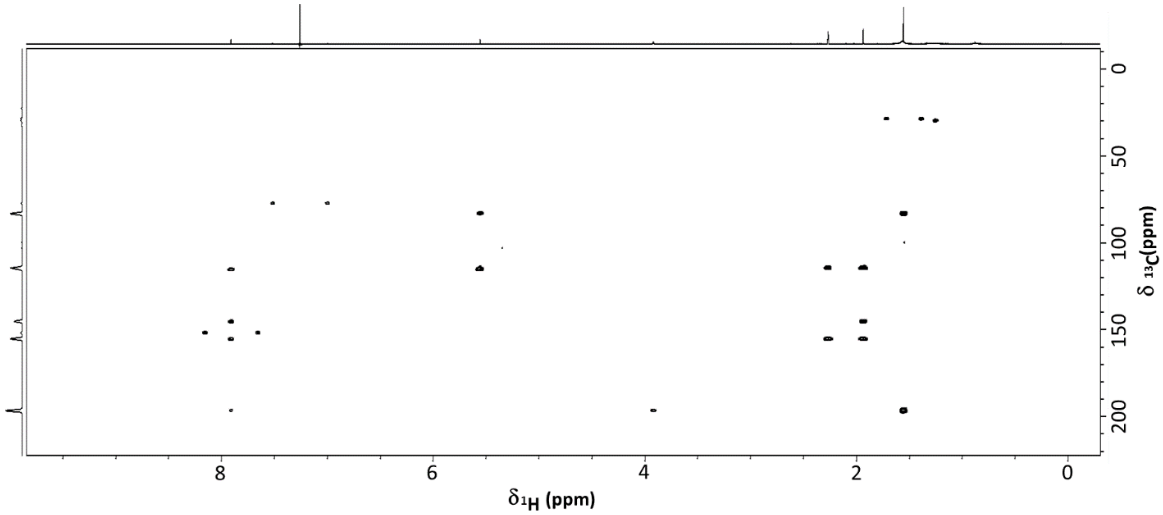

Figure S10. ^{1}H-^{13}C HMBC of chlamyphilone.

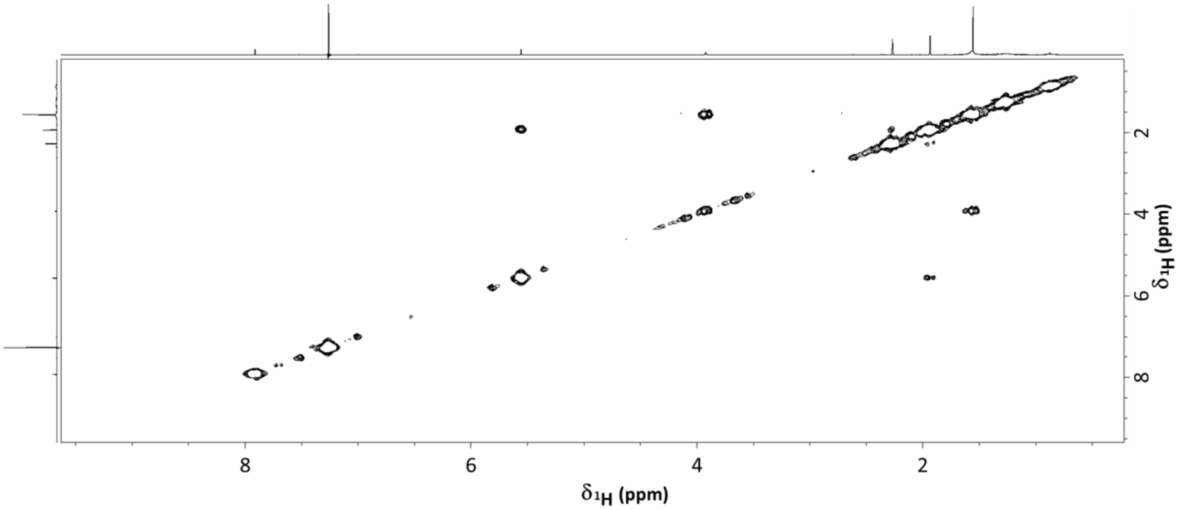

Figure S11. ^{1}H-^{1}H NOESYof chlamyphilone.

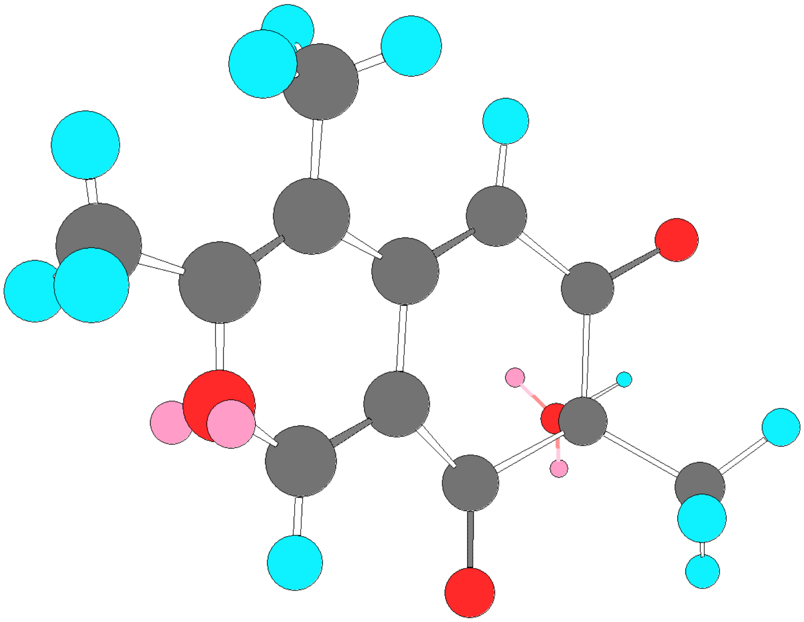

Figure S12. Conformational model of chlamyphilone.

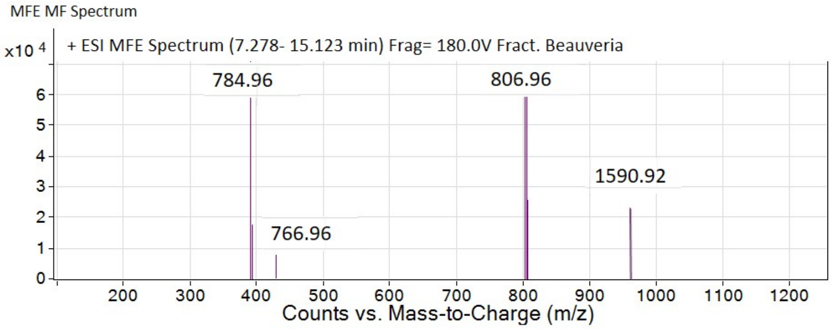

Figure S13. Mass spectrum of fraction 3 from *B. bassiana* corresponding to beauvericin; m/z 784,96 = $[M+H]^+$; m/z 766,96 = $[M+H–H_2O]^+$; m/z 806,96 = $[M+Na]^+$; m/z 1590,92 = $[M_2 + Na]^+$.

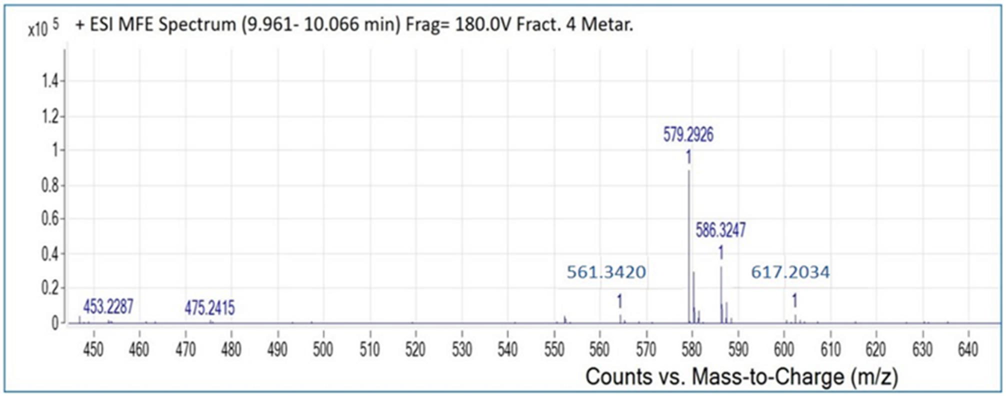

Figure S14. Mass spectrum of Destruxin B2; m/z 579,2926 = $[M+H]^+$; m/z 561,3420 = $[M+H-H_2O]^+$; m/z 617,2034 = $[M+K]^+$.

Table S1: Biological activity of the isolated compounds.

**Fungal species**	**Secondary metabolites**	**% of aphids mortality (72 h after treatment)**	**Biological activities reported on other organisms**	**References***
Paecilomyces lilacinus	Leucinostatins	30 (0.5 mg/ml)	Nematicide (77% of *Caenorhabditis elegans* mortality at 100 mg/L)	Park et al., 2004
Penicillium griseofulvum	Griseofulvin	73 (0.5 mg/ml)	Insecticide (toxic at 250 mg/L in diets on corn earworm, *Helicoverpa zea* Boddie, and on fall armyworm, *Spodoptera frugiperda* (J. E. Smith)	Dowd, 1993
Beauveria bassiana	Beauvericin	40 (0.5 mg/ml)	Insecticide (toxic at different concentration on *Artemia salina,* a model organism to study insecticidal activity, *Calliphora erythrocephala, Aedes aegypti, Lygus spp., Spodoptera frugiperda* and *Schizaphis graminum*)	Wang e Xu, 2012
Metarhizium anisopliae	Destruxin	60 (0.5 mg/ml)	Insecticide on different species (i.e. value for 12-day-old *Spodoptera litura* larvae in combined application assay was 0.045 µg/g body weight, of 0.17 µg/g body weight in the ingestion assay and 0.237 µg/g body weight in the topical application assay)	Soledade et al., 2012; Sree et al., 2008
Talaromyces pinophilus	3-O-Methylfunicone	48 (0.5 mg/ml)	Antibiotic (active at 0.1 mg/mL on *Rhizoctonia solani, Alternaria alternata, Cylindrocladium scoparium* and *Fusariun solani*)	Nicoletti et al., 2009

*

Dowd, P. F. Toxicity of the fungal metabolite griseofulvin to *Helicoverpa zea* and *Spodoptera frugiperda*. *Entomol. Exp. Appl.* **1993**, *69,* 5-11.

Park, J. O.; Hargreaves, J. R.; McConville, E. J.; Stirling, G. R.; Ghisalberti, E. L.; Sivasithamparam, K. Production of leucinostatins and nematicidal activity of Australian isolates of *Paecilomyces lilacinus* (Thom) Samson. *Lett. Appl. Microbiol.* **2004**, *38*, 271276.

Soledade, M.; Pedras, C.; Zaharia, L. I.; Ward, D. E. The destruxins: synthesis, biosynthesis, biotransformation, and biological activity. *Phytochemistry* **2002**, *59,* 579–596.

Sree, K. S.; Padmaja, V.; Murthy, Y. L. N. Insecticidal activity of destruxin, a mycotoxin from *Metarhizium anisopliae* (Hypocreales), against *Spodoptera litura* (Lepidoptera: Noctuidae) larval stages. *Pest Manag. Sci.* **2008**, *64*, 119-125.

Wang, Q.; Xu, L. Beauvericin, a bioactive compound produced by fungi: a short review. *Molecules* **2012**, *17*, 2367-2377.

Nicoletti, R.; Manzo, E.; Ciavatta, M. L. Occurence and bioactivities of funicone-related compounds. *Int. J. Mol. Sci.* **2009**, *10*, 1430-1444.
